# Preferential targeting cancer-related i-motif DNAs by the plant flavonol fisetin for theranostics applications

**DOI:** 10.1038/s41598-020-59343-2

**Published:** 2020-02-13

**Authors:** Shuntaro Takahashi, Snehasish Bhattacharjee, Saptarshi Ghosh, Naoki Sugimoto, Sudipta Bhowmik

**Affiliations:** 1 FIBER (Frontier Institute for Biomolecular Engineering Research), Konan University, 7-1-20 Minatojima-Minamimachi, Chuo-ku, Kobe 650-0047 Japan; 2 Department of Biophysics, Molecular Biology & Bioinformatics, University of Calcutta, University College of Science, 92, A.P.C. Road, Kolkata, 700009 India; 3 FIRST (Graduate School of Frontiers of Innovative Research in Science and Technology), Konan University, 7-1-20 Minatojima-Minamimachi, Chuo-ku, Kobe 650-0047 Japan

**Keywords:** Biophysics, Physical chemistry

## Abstract

The relationship of i-motif DNAs with cancer has prompted the development of specific ligands to detect and regulate their formation. Some plant flavonols show unique fluorescence and anti-cancer properties, which suggest the utility of the theranostics approach to cancer therapy related to i-motif DNA. We investigated the effect of the plant flavonol, fisetin (Fis), on the physicochemical property of i-motif DNAs. Binding of Fis to the i-motif from the promoter region of the human vascular endothelial growth factor (VEGF) gene dramatically induced the excited state intramolecular proton transfer (ESIPT) reaction that significantly enhanced the intensity of the tautomer emission band of Fis. This unique response was due to the coincidence of the structural change from i-motif to the hairpin-like structure which is stabilized via putative Watson-Crick base pairs between some guanines within the loop region of the i-motif and cytosines in the structure. As a result, the VEGF i-motif did not act as a replication block in the presence of Fis, which indicates the applicability of Fis for the regulation of gene expression of VEGF. The fluorescence and biological properties of Fis may be utilised for theranostics applications for cancers related to a specific cancer-related gene, such as VEGF.

## Introduction

**Figure 1:**
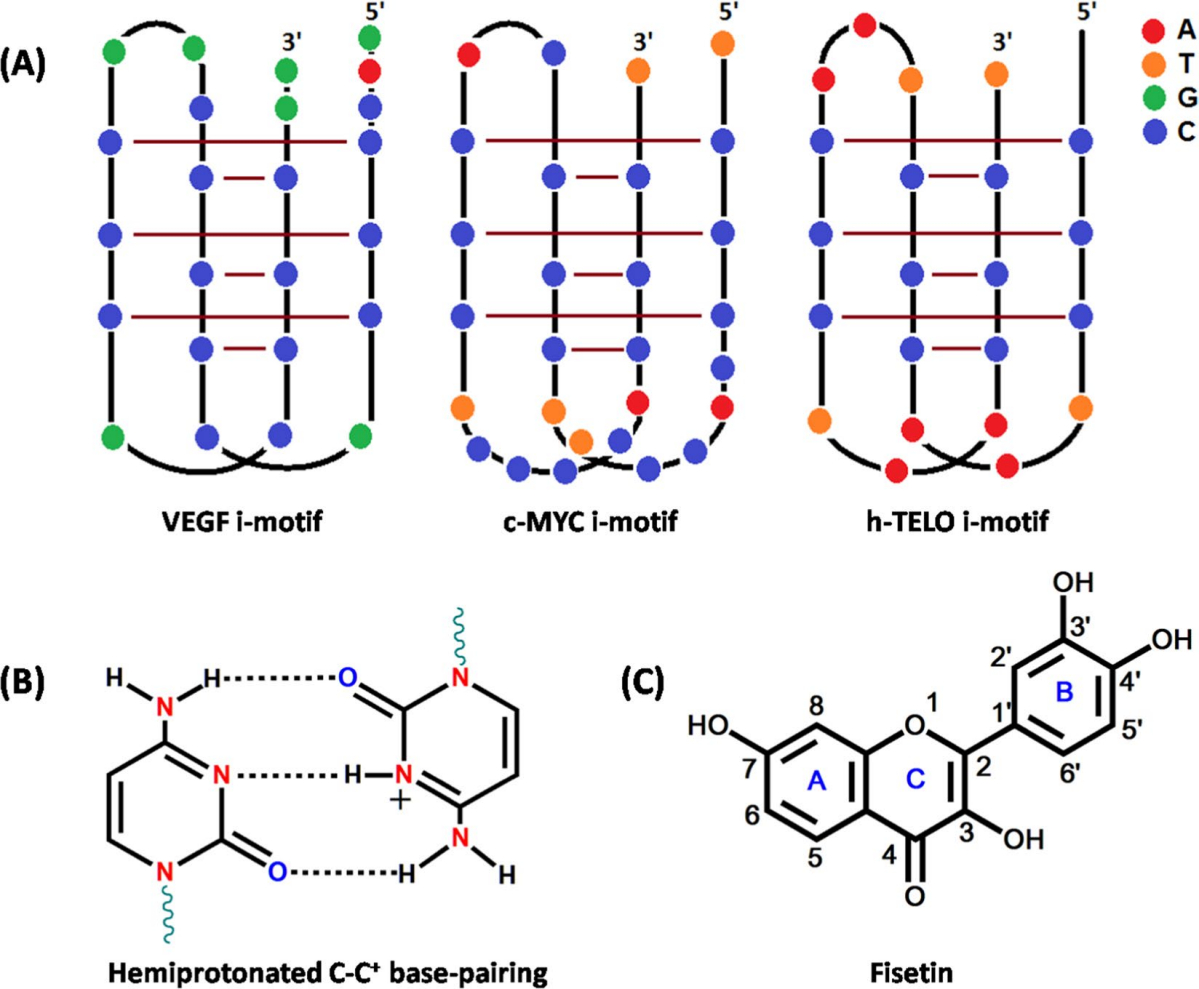
(**A**) Schematic representation of i-motif structures adopted by VEGF, c-MYC, and h-TELO. (**B**) Cytosine–protonated cytosine base pair (C–C^+^). (**C**) Structure of Fisetin (Fis).

Besides the canonical right-handed DNA double helix, DNA sequences can adopt non-canonical structures, including G-quadruplexes (G4s) and i-motifs, that are stabilised by non-Watson-Crick base pairing^1^. G4 structures are formed from guanine (G)-rich sequences, whereas i-motifs are formed from the cytosine (C)-rich sequences. The i-motif structure consists of two parallel duplexes that intercalate with each other in an antiparallel orientation (Fig. 1A). The parallel duplexes are held together via hydrogen bonding between a neutral cytosine (C) and a protonated cytosine (C^+^) (Fig. 1B)^2^. The formation of an i-motif structure occurs more readily in an acidic condition because protonation of cytosine is required to form a hemiprotonated C–C^+^ base pair. However, there is increasing evidence to suggest that the i-motif structures can also form at neutral pH^3,4^ and even in the nuclei of living mammalian cells^5,6^. The i-motif forming sequences are found in or near the promoter regions of >40% of all human genes, which can regulate the expressions of genes like Bcl2^7,8^ and HRAS^9^. Furthermore, the i-motif along the cancer-related DNA sequences strongly inhibits DNA replication^10^. Therefore, i-motif DNAs are attractive targets for the diagnosis of cancer risk and to treat cancer by the modulation of gene transcription and replication. Considering the use of specific targeted therapy based on specific targeted test (i.e., theranostics) for both diagnosis and therapeutics, some ligands that both emit a fluorescent signal upon binding to a specific i-motif and regulate the transcription or replication of the target gene are required. Currently, no ligands have such dual functions for i-motif DNAs.

**Scheme 1:**
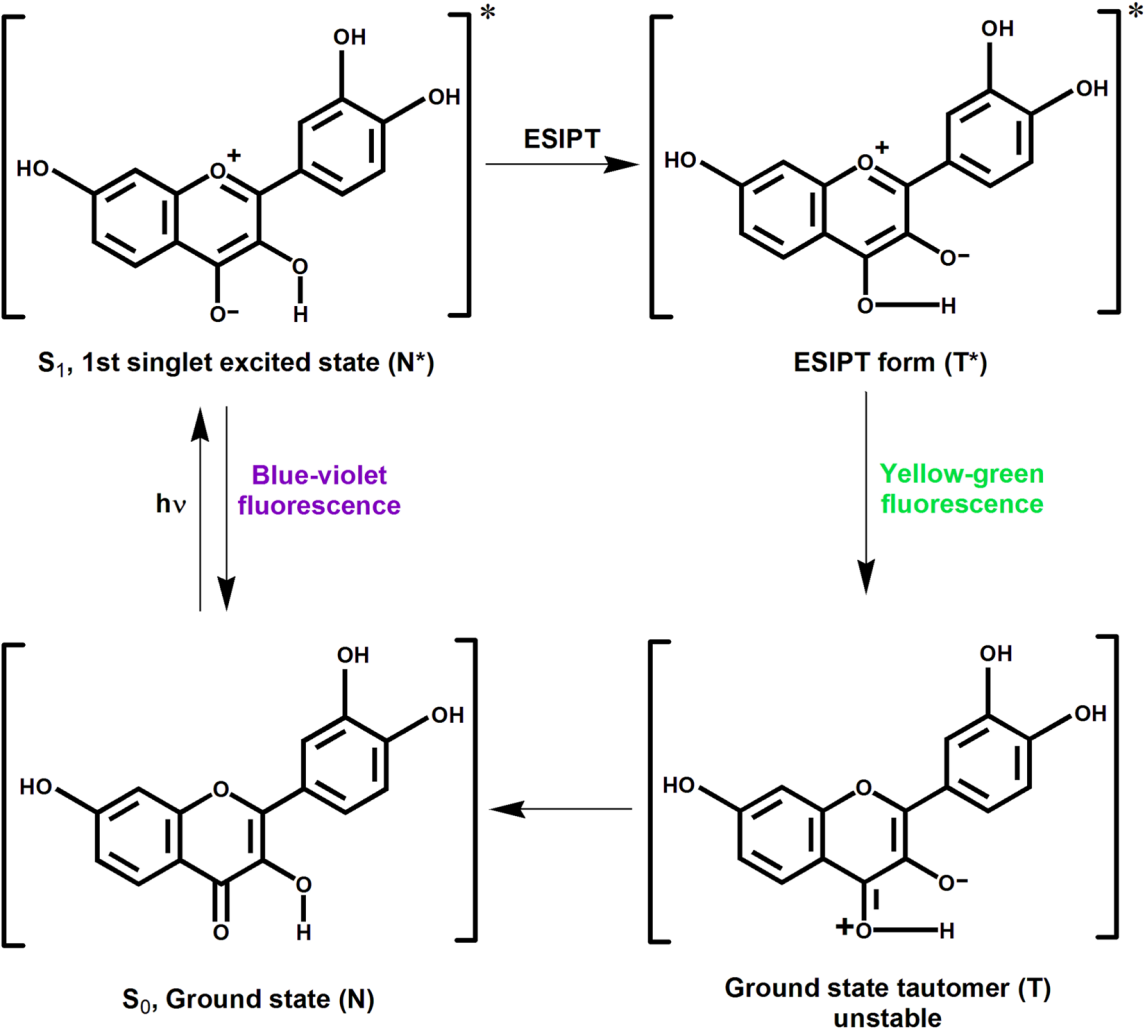
Photoinduced excited state intramolecular proton transfer (ESIPT) in Fis, leading to ‘two colour’ fluorescence behaviour. Ground and excited (indicated by*) states of normal (N) and tautomer (T) forms of Fis.

Flavonols and other related compounds of the flavonoid group are secondary metabolites of plants. They are also small molecule ligands that are very important because of their diverse pharmacological effectiveness that features high potency and low cytotoxicity^11^. Fisetin (Fis; 3,3′,4′,7-tetrahydroxyflavone, Fig. 1C) is a bioactive plant flavonol found in various fruits (strawberries, apples, mangoes, persimmons, kiwis, and grapes), vegetables (tomatoes, onions, and cucumbers), nuts, and wine^12,13^. Fis has attracted attention for its multi-functional therapeutic activities that include anti-cancer^14^, anti-diabetic^15^, and neuroprotective^16^ effects in cell culture and in animal models relevant to human diseases. Fis has a remarkable spectroscopic attribute of an exquisitely sensitive intrinsic ‘two colour’ fluorescence, which arises due to a photoinduced excited state intramolecular proton transfer (ESIPT) reaction (Scheme 1)^17,18^. Previously, our group also explored the preferential interaction of Fis towards G-quadruplex DNA structure^19^. We have also screened various other plant flavonoid molecules such Quercetin, Naringenin, Kaempferol, Morin etc. towards different non-canonical DNA structures such as G-quadruplex and i-motif^19–21^. The gathered experiences over the years working with these classes of small molecules and the multi-functional therapeutic activities of Fis along with the very sensitive fluorescence spectroscopic property inspired us to study its interaction with different and biologically important DNA i-motifs.

In the present work, we investigated the binding of Fis with different i-motif forming DNA sequences from cancer-related DNA sequences present in the promoter region of oncogenes and human telomeres (Table 1 and Fig. 1). Fis exhibited a preferential ESIPT reaction with i-motif from vascular endothelial growth factor (VEGF IM) DNA as compared to those from the promoter region of c-myelocytomatosis (c-MYC IM) and human telomere (h-TELO IM). We also explored the interaction between Fis and variants of VEGF i-motifs (VEGF IM1, VEGF IM2, and VEGF IM3) containing mutations within the loop sequences (Table 1) and found that the guanine bases of the loop regions were essential for the identical ESIPT effect of VEGF IM. Although the stability of i-motif DNA structures did not change upon the binding of Fis, circular dichroism (CD) analysis suggested that Fis transformed VEGF IM to the hairpin-like structure. The observation that Fis released the replication stall at VEGF IM suggests that the processivity of DNA polymerase was recovered due to the preferred replication along the hairpin-like structure. The novel fluorescence and mechanical properties of Fis for the preferred i-motifs may have potential value for cancer theranostics.

**Table 1:** Different i-motif forming sequences used in this study.

Oligoname	Length (bases)	Sequence (5′-3′)
h-TELO IM	22	d(CCCTAACCCTAACCCTAACCCT)
c-MYC IM	28	d(TCCCCACCTTCCCCACCCTCCCCACCCT)
VEGF IM	24	d(GACCCCGCCCCCGGCCCGCCCCGG)
VEGF IM1	24	d(GACCCCCCCCCCCCCGG)
VEGF IM2	24	d(GACCCCCCCCCCCCCCCCGG)
VEGF IM3	24	d(GACCCCGCCCGGCCCGCCCGG)

Modified bases are marked in colour and cytosines involved in i-motif formation are underlined.

## Results

### Spectroscopic changes in Fis upon binding to VEGF IM

It has been reported that the expression of cancer-related genes can be regulated by the formation of an i-motif. Furthermore, replication error may be efficiently caused by the i-motif, which is a possible process of cancer progression. VEGF IM was selected as a target of Fis. Although the biological role of VEGF IM is still unknown, the presence of it in the promoter region of VEGF of a domain in which the i-motif can potentially form is essential for VEGF promoter activity in cancer^22^. The structure of VEGF IM studied by dimethylsulphate (DMS) footprinting was shown to contain six C-C^+^ base pairs in the tetraplex core with three loops consisting of GC, CGG, and GC bases (Fig. 1 and Table 1)^23^.

**Figure 2:**
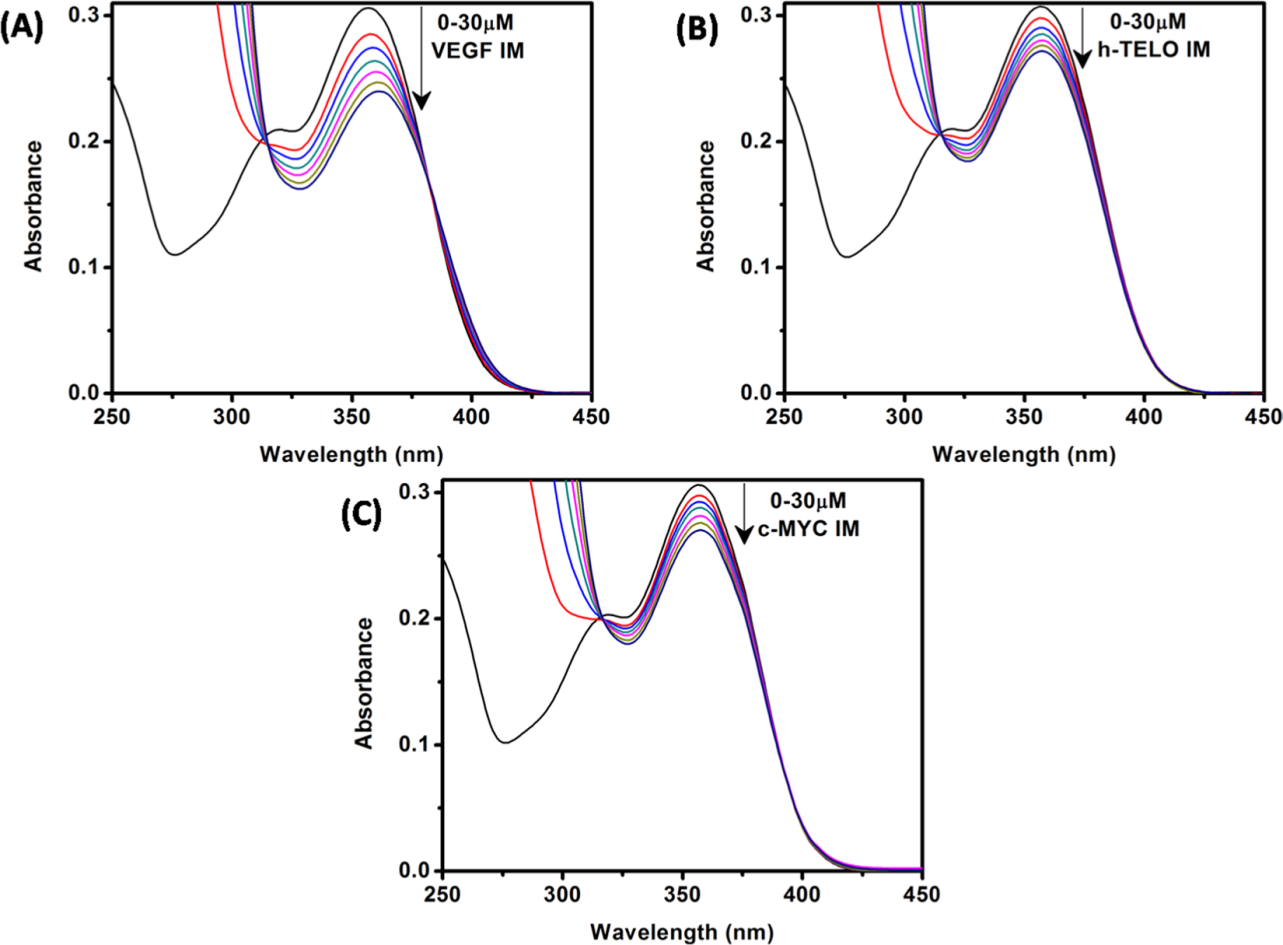
Absorption spectra of Fis (15 μM) in the absence (black curve in each case) and presence of successive additions of VEGF IM (**A**), h-TELO IM (**B**), and c-MYC IM (C) DNA. All the experiments were performed in a buffer consisting of 50 mM KCl, 10 mM KH_2_PO_4_, and 1 mM K_2_EDTA (pH 5.4) at 25 °C.

We first performed UV-vis absorption analysis to explore the binding interaction between VEGF IM and Fis. In general, the absorption profile of Fis showed two absorption bands (band I and band II). Band I located in the wavelength range of 300–400 nm is attributed to the light absorption of the cinnamoyl group (B + C ring) whereas band II located in the wavelength range of 240–300 nm is related to the absorption of the benzoyl group (A + C ring) (Fig. 1C)^24,25^. The lowest energy absorption maximum of Fis was observed at approximately 356 nm in the aqueous buffer solution at pH 5.4, which agreed with previous reports^26,27^. In the presence of 30 μM VEGF IM, the absorption spectra of Fis displayed decreased absorbance (21.6% hypochromism) along with a 5 nm bathochromic (red) shift of the absorption maximum (Fig. 2A). The reduced absorption along with red shift of the absorption maxima inferred a possible switch of polarity around the Fis molecules due to the binding interaction with VEGF IM. For the c-MYC IM and h-TELO IM structures, the extent of hypochromism was less compared to VEGF IM (11.6% and 11.5% hypochromism, respectively), with no observable spectral shift of Fis (Fig. 2B,C). These results clearly demonstrated the potential for interaction of Fis with VEGF IM compared to c-MYC IM and h-TELO IM.

**Figure 3:**
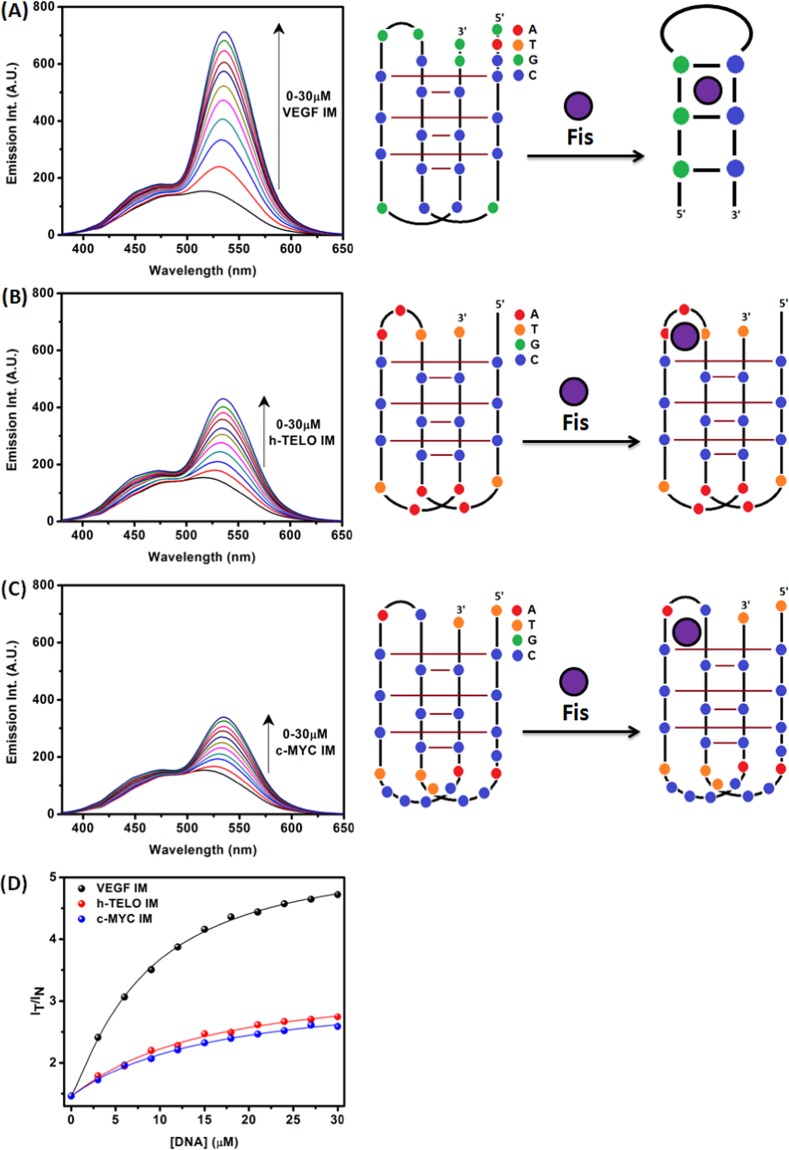
Fluorescence emission spectra of Fis (10 μM) with increasing concentrations of VEGF IM (**A**), h-TELO IM (**B**), and c-MYC IM (**C**) DNA [λ_ex_ = 365 nm]. Figure (**D**) represents variation of the I_T_/I_N_ (I_535nm_/I_450nm_) values for Fis with increase in concentration of VEGF IM (black), h-TELO IM (red) and c-MYC IM (blue). All the experiments were performed in a buffer consisting of 50 mM KCl, 10 mM KH_2_PO_4_, and 1 mM K_2_EDTA (pH 5.4) at 25 °C.

Next, the fluorescence property of Fis upon binding of VEGF IM was investigated. In the aqueous medium, the fluorescence emission spectrum of Fis appeared as a broad emission band due to an overlap of normal and tautomer emission bands^17,18^. The addition of VEGF IM induced dramatic changes in the emission behaviour of Fis (Fig. 3A). Upon the addition of VEGF IM, conspicuous dual fluorescence was noted, with the appearance of a strong yellow-green tautomer fluorescence band with a maximum at approximately 540 to 545 nm, and a blue-violet normal emission with a maximum at approximately 490 to 500 nm) (Fig. 3A). The blue-violet fluorescence band was assigned to the S_1_ (ππ*) → S_0_ normal (N*, non-proton-transferred) emission and the large Stokes shifted yellow-green fluorescence band was assigned to emission from the tautomer species (T*) generated by an ESIPT process from the C(3)–OH to the C(4)=O of the Fis molecules (Scheme 1)^17,18^. The observed enhanced tautomer emission indicated that in the presence of VEGF IM, the Fis molecules experience relatively hydrophobic environments where the external hydrogen bonding interference to ESIPT is minimised, and the ESIPT process is facilitated. The ratio of the intensities of the tautomer (I_T_; 535 nm) and the normal band (I_N_; 450 nm) emissions (I_T_/I_N_), which is a useful parameter to quantitatively understand the microenvironment of Fis^19,28–31^, was approximately 4.8 for 30 μM VEGF IM. The fluorescence spectra of Fis in the presence of c-MYC IM and h-TELO IM are shown in Fig. 3B,C, respectively. I_T_/I_N_ of Fis in the presence of 30 µM of c-MYC IM and h-TELO IM were 2.59 and 2.74, respectively. The I_T_/I_N_ of Fis in the presence of VEGF IM was high as compared to the other i-motif DNAs (c-MYC IM and h-TELO IM). From the DNA concentration dependency of the ratio (I_T_/I_N_), we estimated the binding constants of Fis with VEGF IM as *K*_a_ = 9.8 × 10^4^ M^−1^ at 25 °C. This value was slightly higher in comparison to other i-motif DNAs: c-MYC IM (*K*_a_ = 5.8 × 10^4^ M^−1^) and h-TELO IM (*K*_a_ = 5.9 × 10^4^ M^−1^) at 25 °C (Supplementary Information, Fig. S1). We observed the binding constant values in the order of 10^4^ M^−1^, which agreed well with the literature for flavonol-DNA interactions^28^.

### Structural transformation of VEGF IM upon binding of Fis

**Figure 4:**
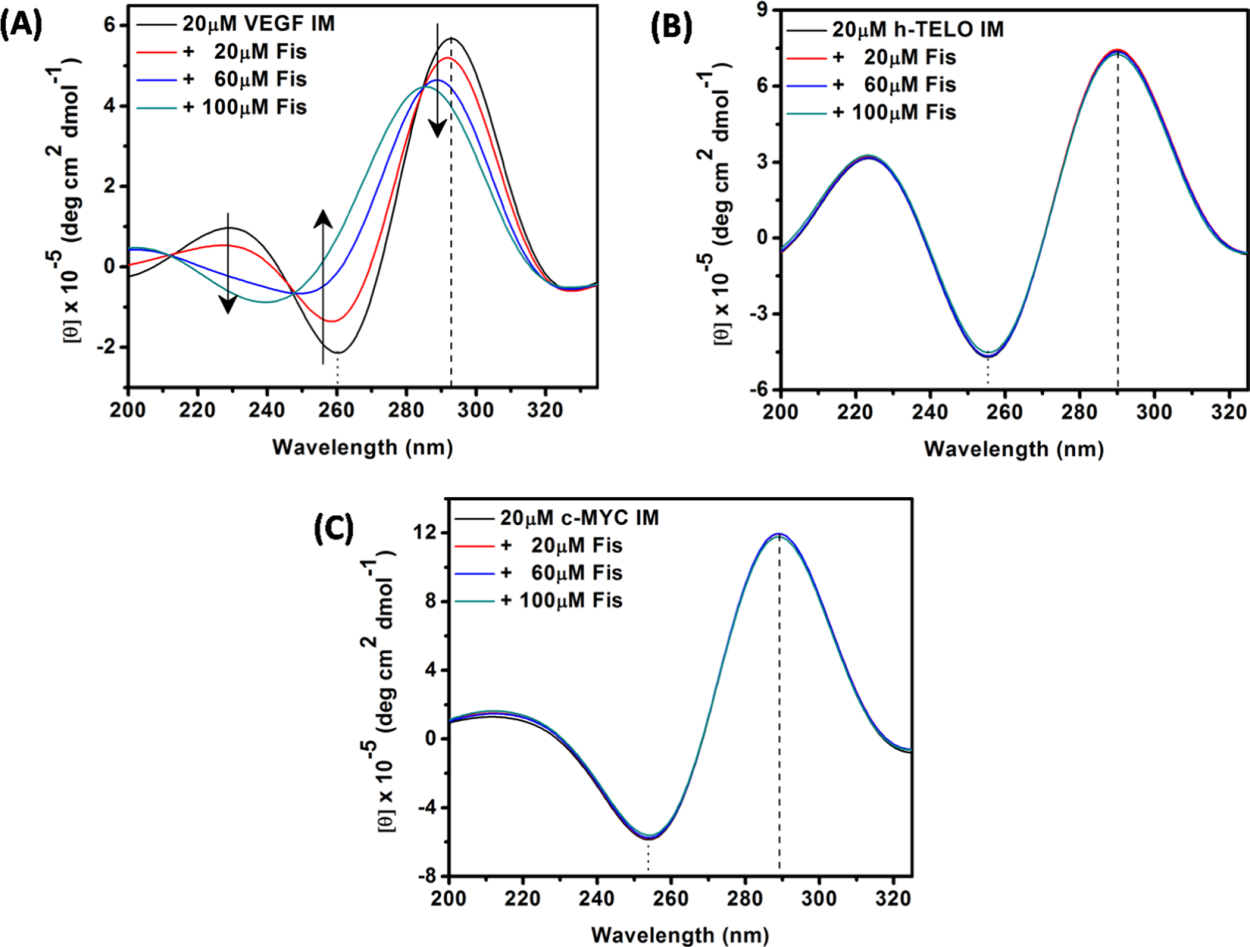
CD spectra of VEGF IM (**A**), h-TELO IM (**B**), and c-MYC IM (**C**) DNA in the absence (black spectrum in each case) and presence of different concentrations of Fis. All the experiments were performed in a buffer consisting of 50 mM KCl, 10 mM KH_2_PO_4_, and 1 mM K_2_EDTA (pH 5.4) at 25 °C.

To investigate the effect of Fis on the conformation of VEGF IM, we analysed the structure of the complex of VEGF IM and Fis by CD spectroscopy. In the absence of Fis, VEGF IM exhibited a positive band centred at approximately 290 nm and a negative band at approximately 260 nm (Fig. 4), which are the characteristic bands of i-motif structures^32^. Upon the addition of Fis to VEGF IM, we observed drastic changes in the CD profile, manifested by significant changes in the band position and band intensity. These findings indicated a strong perturbation of the i-motif DNA structure (Fig. 4A). In the presence of 100 µM Fis, 20 µM VEGF IM displayed a positive band at approximately 282 nm and a negative band at approximately 240 nm. This was a similar characteristic to the duplex and hairpin structures^32^. Several studies have reported the transformation of an i-motif to a hairpin-like structure^7,33^. On the other hand, the CD spectra of c-MYC IM (Fig. 4B) and h-TELO IM (Fig. 4C) did not show such dramatic changes upon the addition of Fis.

To assess the physical property of the complex of VEGF IM and Fis, the UVmelting experiment was done to identify the thermal stability of the structures. As shown in Supplementary Fig. S2, melting profiles measured at 260 nm showed no significant difference in melting temperature (*T*_m_) of VEGF IM upon the addition of Fis. The change in pH of the phosphate buffer with increasing temperature has been monitored and a slight increase of pH from 5.40 to 5.57 with increasing temperature from 20 ^o^C to 40 ^o^C was observed. Whereas, the pH of the buffer slightly decreased from 5.57 to 5.36 with increasing temperature from 40 ^o^C to 90 ^o^C. Thus, it is evident that the observed pH change did not affect the i-motif structure (Fig. S3)^34,35^. The absorbance of 50 µM Fis at 260 nm was analyzed over the entire temperature range and no significant change in the absorbance of Fis was observed which can affect the melting profile of i-motif (Fig. S4). These results indicated that the binding of Fis can transform VEGF IM to the hairpin like structure without altering the stability of the structure. VEGF IM contains GC-rich sequence and binding of Fis can transform VEGF IM to the hairpin duplex in which the two strands may stabilize by the G-C base pairing. Thus it may be the reason that although the structural change is occurring but there is no change in the *T*_m_ of VEGF IM upon addition of Fis, suggesting the fact that both forms have almost identical stability. Probably, few G-C base pairing in the hairpin structure compensated the overall stability of the hydrogen bonding between neutral cytosine (C) and protonated cytosine (C^+^) in the i-motif structure. This finding, regarding the no change in stability of VEGF IM upon interacting with Fis, showed similar trend with the other reported i-motif based fluorescence ligands^36,37^.

### Effects of loop sequence of VEGF IM on its structural transformation by Fis

**Figure 5:**
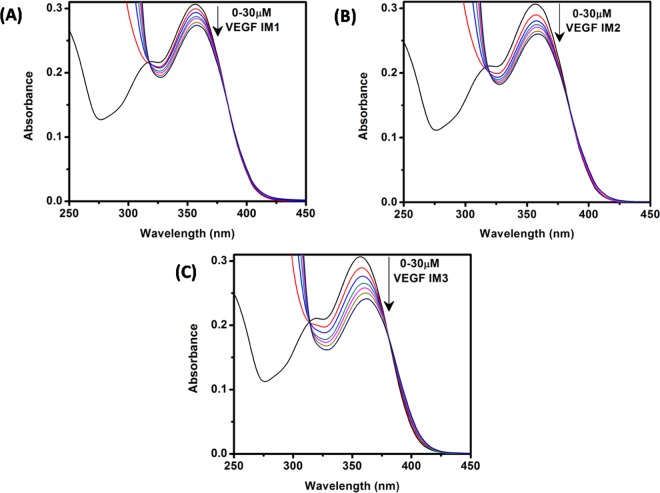
Absorption spectra of Fis (15 μM) in the absence (black curve in each case) and presence of successive additions of VEGF IM1 (**A**), VEGF IM2 (**B**), and VEGF IM3 (**C**) DNA. All the experiments were performed in a buffer consisting of 50 mM KCl, 10 mM KH_2_PO_4_, and 1 mM K_2_EDTA (pH 5.4) at 25 °C.

To investigate the mechanism of the structural change inVEGF IM by Fis, we mutated the loop region of VEGF IM (VEGF IM1, VEGF IM2, and VEGF IM3; see Table 1). Since the CD studies indicated that VEGF IM, c-MYC IM, and h-TELO IM form identical anti-parallel structures, we postulated that the differences in loop sequences may be responsible for the preferential interaction of Fis with VEGF IM. The absorption spectroscopy data revealed a reduced interaction of Fis in the case of VEGF IM1 (10.8% hypochromism; no red shift) and VEGF IM2 (15.2% hypochromism; 2 nm red shift). However, the extent of interaction with VEGF IM3 (22.6% hypochromism; 5 nm red shift) was almost the same compared to the wild-type VEGF IM sequence (21.6% hypochromism; 5 nm red shift)(Fig. 5).

**Figure 6:**
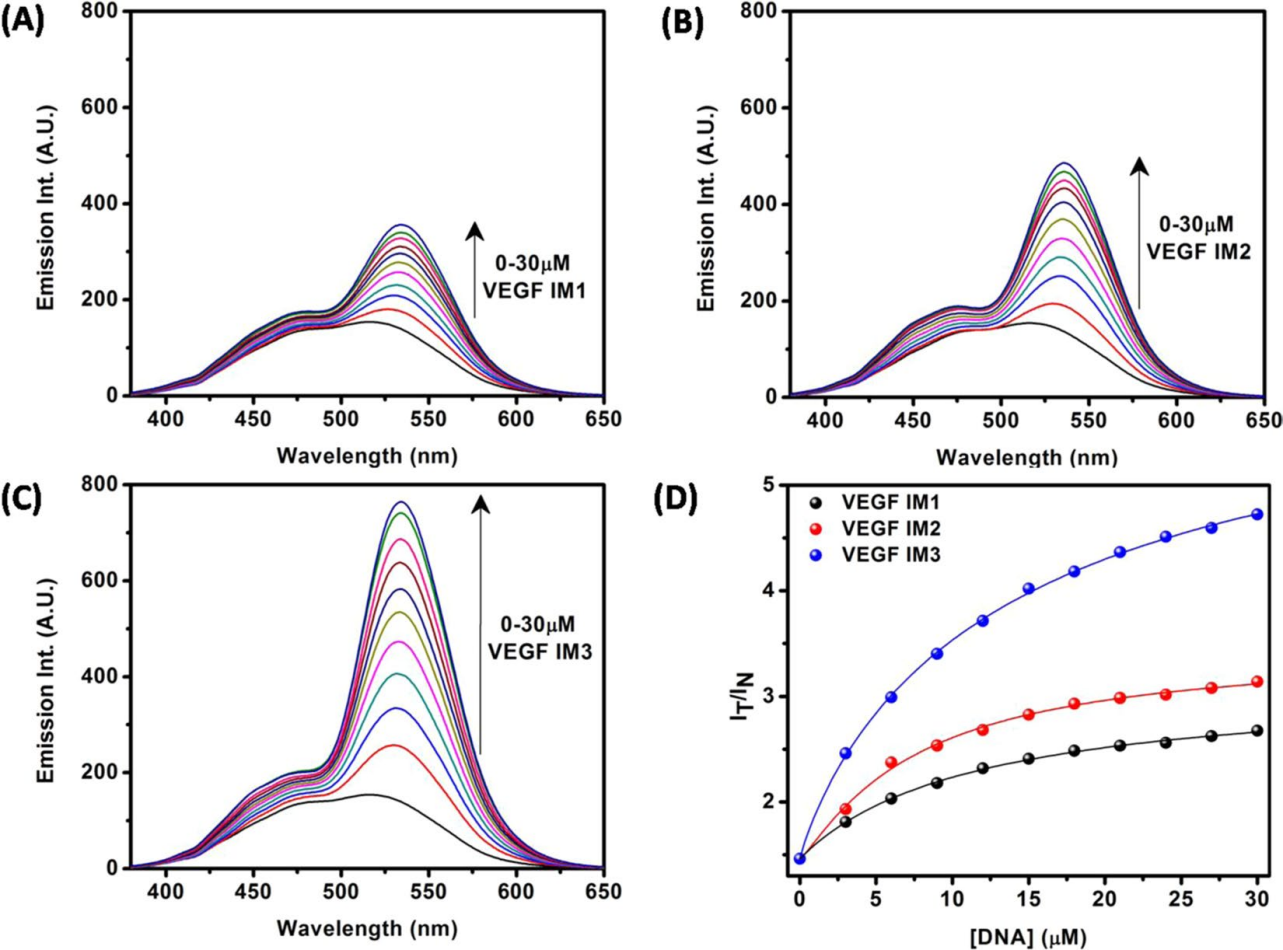
Fluorescence emission spectra of Fis (10 μM) with increasing concentrations of VEGF IM1 (**A**), VEGF IM2 (**B**), and VEGF IM3 (**C**) DNA [λ_ex_ = 365 nm]. Figure (**D**) represents variation of the I_T_/I_N_ (I_535nm_/I_450nm_) values for Fis with increase in concentration of VEGF IM1 (black), VEGF IM2 (red) and VEGF IM3 (blue). All the experiments were performed in a buffer consisting of 50 mM KCl, 10 mM KH_2_PO_4_, and 1 mM K_2_EDTA (pH 5.4) at 25 °C.

The fluorescence property of Fis upon binding with the modified VEGF IMs was investigated. Both I_T_/I_N_ (Fig. 6) and *K*_a_ (Supplementary Information, Fig. S1) values were higher in the case of VEGF IM3 compared to those in VEGF IM1 and VEGF IM2. The observed *K*_a_ values were 6.2 × 10^4^ M^−1^ for VEGF IM1, 6.9 × 10^4^ M^−1^ for VEGF IM2, and 8.6 × 10^4^ M^−1^ for VEGF IM3 at 25 °C. The findings indicated the preferential interaction of Fis with VEGF IM3 among the modified sequences, with the extent of interaction being nearly the same as with wild-type VEGF IM.

**Figure 7:**
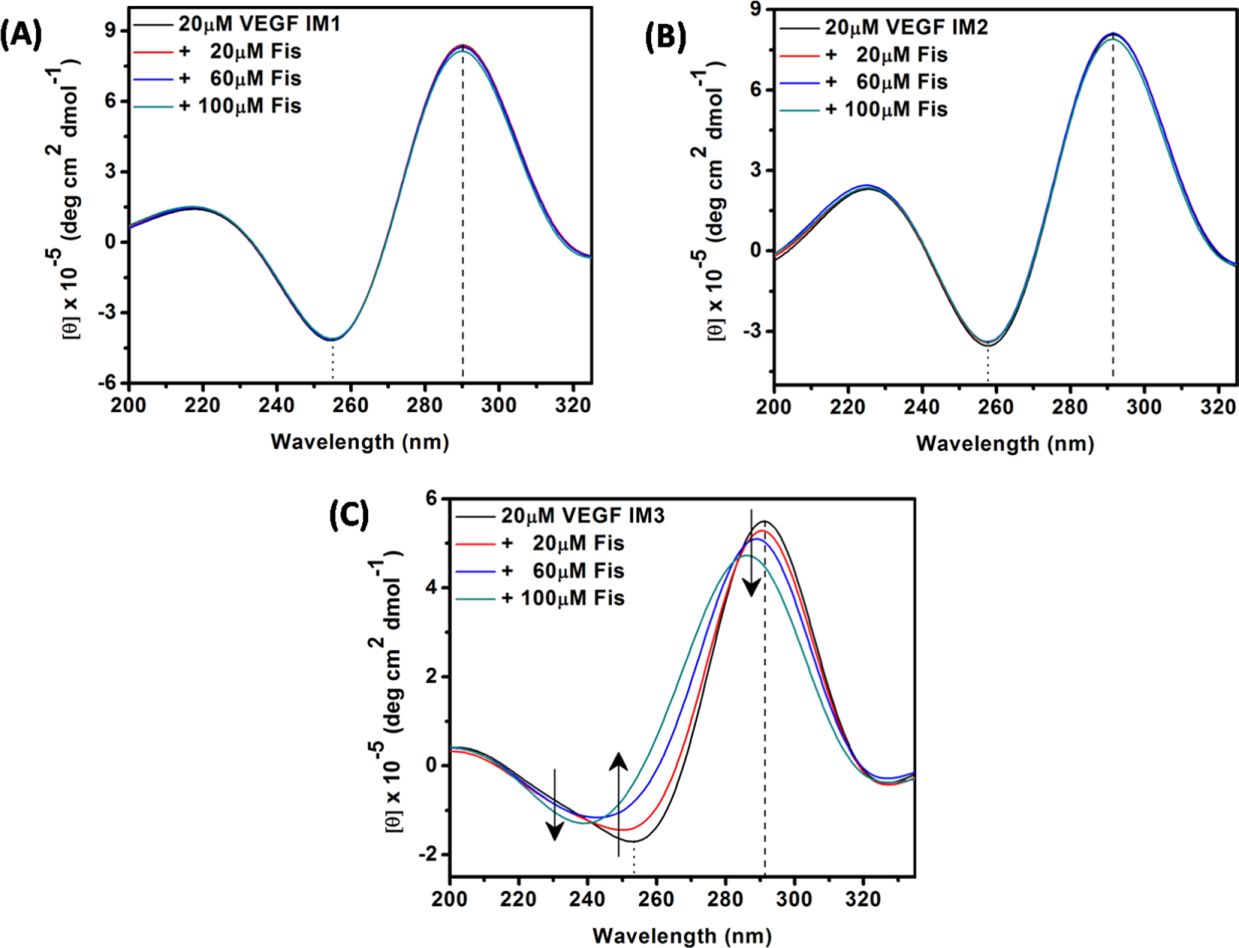
CD spectra of VEGF IM1 (**A**), VEGF IM2 (**B**), and VEGF IM3 (**C**) DNA in the absence (black spectrum in each case) and presence of different concentrations of Fis. All the experiments were performed in a buffer consisting of 50 mM KCl, 10 mM KH_2_PO_4_, and 1 mM K_2_EDTA (pH 5.4) at 25 °C.

These spectroscopic results echoed the CD results (Fig. 7). In the absence of Fis, the modified VEGF IMs exhibited characteristic bands of i-motif structures, indicating the formation of these structures. VEGF IM1 and VEGF IM2 (both 20 µM) did not show any different bands upon the addition of up to 100 µM of Fis (Fig. 7A,B). On the other hand, 20 µM VEGF IM3 showed structural transition by the addition of Fis up to 100 µM, similar to the wild-type structure of VEGF IM (Figs. 4A and 7C).

UV melting was used to identify the thermal stability of the mutated VEGF i-motif structures upon binding with Fis. As shown in Supplementary Fig. S2, Fis had no effect on the thermal stability of mutated VEGF i-motifs.

The above results indicate that the presence of guanine bases in the loop region of VEGF IM is important for the interaction of Fis as well as for the transformation of i-motif structure to hairpin.

### Replication control of VEGF IM DNA by the addition of Fis

From the therapeutic view point, we investigated whether the structural transformation by Fis influenced the replication reaction. Previously, we reported that the replication reaction can be affected by the formation of non-canonical structures on the template DNA and that the i-motif especially blocked the processivity of DNA polymerase efficiently^10^. For the replication analysis, a fluorescein-labelled primer and template DNA containing VEGF IM was designed as reported previously^10^. The replication reaction was carried out using these DNAs and Klenow fragment DNA polymerase lacking 3′ → 5′ exonuclease activity (hereafter: KF exo-) in the absence or presence of Fis at 37 °C and pH 6.0. The efficiency of the replication stall of the template DNA at the i-motif was analysed by denaturing polyacrylamide gel electrophoresis (PAGE) in reaction samples obtained at each time point.

**Figure 8:**
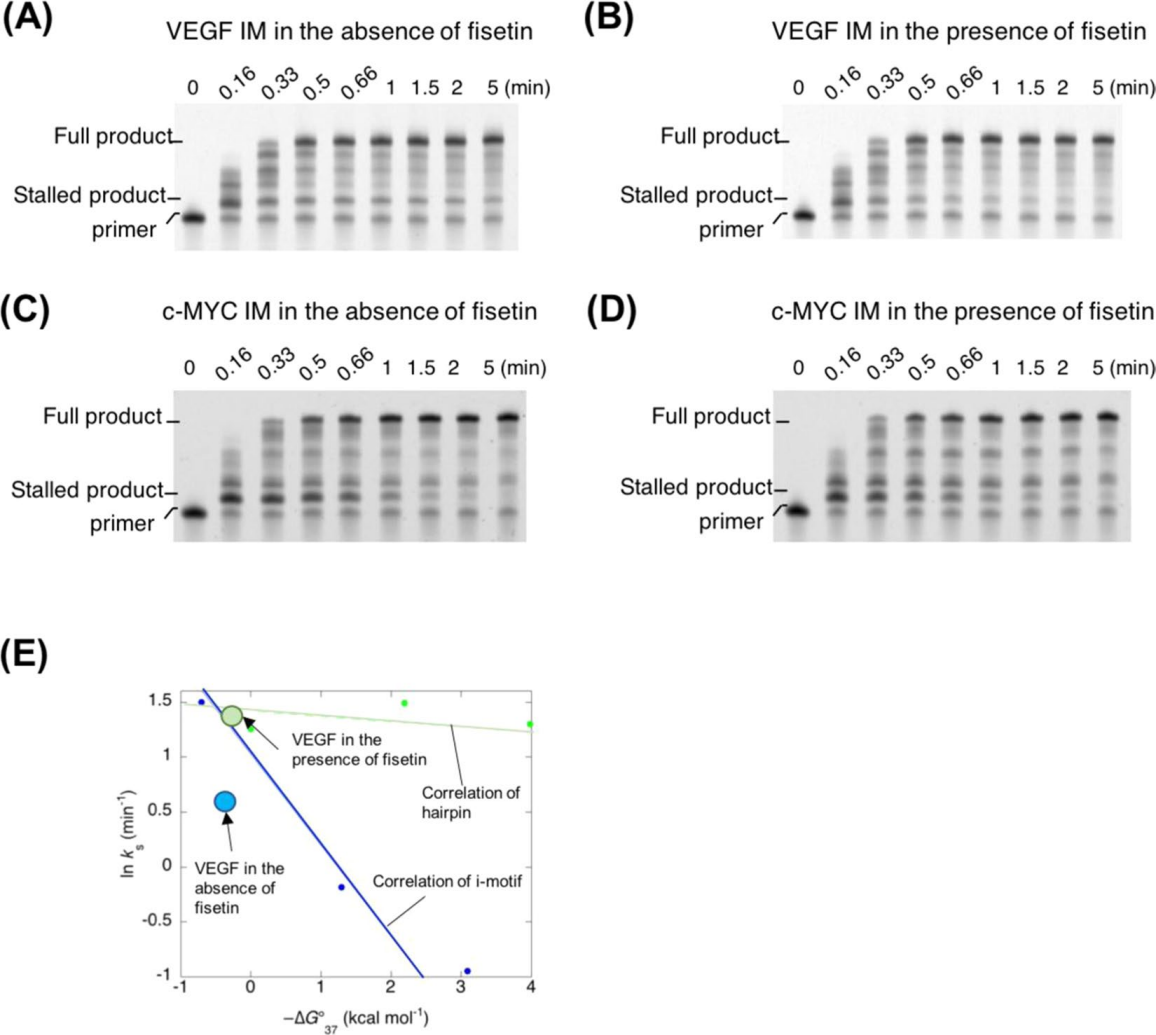
Replication assay of i-motif containing sequence having (**A**) VEGF IM without Fis, (**B**) VEGF IM with 100 µM Fis, (**C**) c-MYC IM without Fis, and (**D**) c-MYC IM with 100 µM Fis. (**E**) QSTR plot of −∆*G*°_37_ values vs. ln*ks* for reactions to dissolve the stall from reaction start along VEGF IM with or without Fis. The linear lines indicate i-motif–forming templates (blue) and hairpin forming template (green). All the replication assays were carried out in 40 mM MES (pH 6.0), 50 mM KCl, 8 mM MgCl_2_, 1 μM KF exo-, 1 μM DNAs, and 250 μM dNTPs at 37 °C.

In the absence of Fis, two major products were observed. The longer product represented the fully replicated product from the template DNA and the shorter product represented KF exo-stalled immediately before the VEGF IM formed on the template DNA. The short product obtained from stalled complexes was observed in reactions containing the VEGF IM sequence (Fig. 8A). The stalled products were evident as soon as 5 min after starting the replication reaction. On the other hand, in the presence of 100 µM Fis, the replication product showed very low amounts of stalled products and immediate accumulation of full-length products (Fig. 8B). The stalled products were nearly undetectable 1 min after the reaction. The efficiency of replication depends on the stability and the topology of the non-canonical structure on the template DNA^10,38^. Furthermore, our previous study suggested that a hairpin structure produces less inhibition of replication than the i-motif^10^. As observed in the UV melting analysis, the stability of VEGF IM did not increase in the presence of Fis, whereas the structural change from the i-motif to the hairpin-like form was estimated by the CD analysis. Thus, the change in the replication efficiency of VEGF IM could be due to the structural change in the i-motif induced by the Fis binding. Indeed, the control of Fis replication was not observed in the case of the template DNA having c-MYC IM (Fig. 8C,D), which did not show the structural change by Fis. We performed a quantitative study of topology-dependent replication (QSTR) analysis^10,38^. The time course of the full-length product formation was tracked to determine the rate constants at 37 °C for overcoming the i-motif structure (*k*_s_; Supplementary Information, Fig. S5). The QSTR plot revealed the relationship between ln*k*_s_ and −∆*G*°_37_ (Fig. 8E). The replication of the VEGF IM in the absence of Fis had a *k*_s_ of 1.8 min^−1^ at 37 °C. On the other hand, the *k*_s_ value in the presence of Fis was estimated as 3.9 min^−1^. Thermodynamic analysis of the UV melting profiles revealed a −∆*G*°_37_ of −0.35 kcal mol^−1^ and −0.25 kcal mol^−1^ for VEGF IM in the absence of Fis and in the presence of 100 µM Fis, respectively. Figure 8E shows the QSTR plots of VEGF IM in the absence and presence of Fis together with the linear correlation of i-motifs and hairpins determined previously^10^. This relationship indicated that the QSTR plots for hairpin DNAs increased compared to those for i-motif DNAs, indicating that the replication of hairpin DNAs proceeded more efficiently than that of i-motif DNAs. Interestingly, the plot of VEGF IM in the absence of Fis revealed a downward trajectory of the linear correlation of i-motifs, whereas that in the presence of Fis the plot resembled that of hairpins. These findings suggested that VEGF IM can act as an i-motif or hairpin to control replication induced by the Fis binding. Therefore, the transition from i-motif to hairpin structure by Fis would be effective to prevent replication from pausing aberrantly.

## Discussion

The involvement of i-motifs has been demonstrated in the transcription control of several cancer-related genes. The strategy for design of compound that bind to the i-motif has become a topic of intensive research interest for cancer diagnosis and therapeutics. Carboxyl-modified single-walled carbon nanotubes (SWCNTs) are considered the first selective i-motif ligands, as well as inducers of i-motif formation under physiological conditions^39^. In 2012, Qu *et al*. investigated the biomedical effect of C-SWNTs on telomerase activity and telomere function and it was reported that the presence of C-SWNTs could inhibit telomerase activity, interfere with telomere functions, and lead to senescence and apoptosis in cancer cells^40^. The possible targets of drugs in i-motif structures are terminal C:C^+^ base pairs, grooves, and loops. Since i-motifs have different categories based on the topology and the loop length, it is possible to find or design a structure-specific i-motif that is the target of binding^41^. Various ligands including berberine^42^ and thioflavinT^43^ could bind to the groove or the cavity in loops of i-motifs and emit fluorescence. It has been suggested that Fis binds to the loop region of i-motif^28^. Presently, Fis displayed strong fluorescence due to the enhanced ESIPT signal when it bound to all the i-motifs tested, especially VEGF IM. Although the enhancement of ESIPT of Fis by the i-motif has been known^28^, the present data highlight the sequence-dependent manner of fluorescence from Fis upon binding to the i-motif from the VEGF gene. Considering that Fis transformed VEGF IM to the hairpin-like structure, Fis might bind to the groove of c-MYC IM and h-TELO IM, where it could be specifically captured in the more hydrophobic space created by the hairpin-like structure than that in the groove of i-motifs.

The precise information about the structure of the hairpin-like structure is not yet known. However, it has been reported that a hairpin-like structure can form from the C-rich strand of C9ORF72 consisting of a C_4_G_2_ repeat^44^. This hairpin structure is formed with Watson-Crick base pairing of the cytosine and guanine bases, and may further fold into a tertiary structure. Both VEGF IM and the i-motif of C9ORF72 have several guanines in the loop, which implies that VEGF IM with Fis might form a hairpin structure similar to that of C9ORF72. Since the number of guanines within the loop of VEGF IM is smaller than that of C9ORF72, the stability of the hairpin structure of VEGF IM should be decreased compared to C9ORF72 and stability should be increased by the interaction with Fis.

IMC-76 regulates gene expressions via i-motifs. This involves the specific induction of the hairpin form of the promoter region of the Bcl2 C-rich strand, which results in the repression of transcription^7^. As observed in the replication assay, Fis also can regulate biological reactions by the induction of the hairpin conformer of the promoter region of the VEGF C-rich strand. Thus, Fis can be used for diagnosis (sensing of the structure) and for therapeutics (regulation of the structure). Although these ligands that induce a hairpin from the i-motif share the biological effect of the regulation of gene expressions, Fis does not induce the hairpin transformation of the Bcl2 i-motif (Supplementary Information, Fig. S6). This result suggests that Fis has high specificity for VEGF IM likewise IMC-76 for the Bcl2 i-motif. The structural difference of these i-motifs is the category of class—VEGF IM is categorized in the class I i-motif having short loops, and Bcl2 into class II having long loops. In the case of Bcl2, the loop region is relatively long, but has a small number of guanines compared to VEGF IM. Thus, IMC-76 likely binds to the hairpin structure in a sequence-specific manner. On the other hand, VEGF IM has short loops and few variations to provide a sequence-specific site for the ligand with its hairpin form. Therefore, as discussed above, Fis might prefer the hairpin-like structure from the class I i-motif that folds into a tertiary complex structure. To understand the importance of loop sequences in the VEGF IM, we have mutated the loop region (Table 1). The findings clearly indicated that the presence of guanine bases in this short loop region is necessary for the transformation of VEGF IM to hairpin structure by the Fis (Figs. 5–7). Further structural analyses at the atomic level are expected to reveal the structure, which will inform the design of novel drugs that distinguish between the structures of i-motifs.

Much effort is focused on the development of compounds that bind to non-canonical structures, such as G4 motifs and i-motifs. Many compounds have been identified. Some stabilize structure and result in emission of fluorescence. Thus, these drugs can induce the structure of target sequences. However, although this type of binder is a promising drug, it may induce an unnecessary non-canonical structure, which could lead to the misdiagnosis of the formation of the non-canonical structures and cause side effects by the perturbation of gene expression. On the other hand, Fis does not induce stabilisation of the i-motif structure but transforms it to the hairpin-like structure, which is a superior property for a theranostic drug.

In summary, the natural dietary plant flavonol Fis is able to bind a VEGF i-motif forming DNA sequence preferentially over other i-motif DNAs of similar structural topology. The interaction of Fis with VEGF IM caused both emission of tautomer fluorescence and transformation of the i-motif structure to the hairpin-like structure. The former property can be used for detection of the specific i-motif structure and is thus applicable to the diagnosis of aberrant formation of i-motifs, such as VEGF and other i-motif having guanine residues within the loops of the i-motif. The latter facilitates replication of these i-motif forming regions, where the i-motifs hinder the processivity of DNA polymerase. As this hindrance can be a cause of diseases like cancers, the control of replication by Fis is also therapeutically important and feasible. Fis may act as a novel theranostic drug. This study provides useful information for the development of i-motif based drug targets and offers a novel approach for the development of drugs targeting non-canonical structures for theranostics.

## Methods

### Materials

Different i-motif forming DNA sequences (Table 1) and the flavonol Fis were purchased from Sigma-Aldrich, and were used as received. The solvents used were of spectroscopic grade. The buffer solution used here to form the i-motif structure contains 50 mM KCl, 10 mM KH_2_PO_4_, and 1 mM K_2_EDTA (pH 5.4). The DNA was annealed according to the procedure described in Bhattacharjee *et. al*.^21^. Stock solution of Fis was prepared in methanol and the final experimental concentrations of Fis were kept in the micromolar range in <1% (v/v) methanol.

### Ultraviolet-visible absorption analysis

Ultraviolet-visible (UV-Vis) absorption spectra of Fis were recorded with a V-630 spectrophotometer using a 1-cm path length quartz cuvette. Fis concentration was kept constant at 15 µM and titrated with increasing concentrations(0–30 µM) of different i-motif DNAs. All the UV-Vis absorption measurements were carried out in buffer containing 50 mM KCl, 10 mM KH_2_PO_4_, and 1 mM K_2_EDTA (pH 5.4) at 25 °C.

### Fluorescence spectroscopy analysis

The fluorescence emission spectra were recorded with an FP-8500 fluorescence spectrometer (Jasco International Co. Ltd.) using a quartz cuvette with a 1-cm path length. During emission spectral measurements both the excitation and emission spectral slit widths were set to 5 nm, and the excitation wavelength for Fis was set at 365 nm. Experiments were carried out by keeping the concentration of Fis constant (10 µM) and titrated with increasing concentrations (0–30 µM) of different i-motif DNAs. Fluorescence studies were carried out using buffer containing 50 mM KCl, 10 mM KH_2_PO_4_, and 1 mM K_2_EDTA (pH 5.4) at 25 °C. The ratio of the intensities of the tautomer (I_T_; 535 nm) and the intensities of normal band emissions (I_N_; 450 nm) of Fis titration data^26^ were used to determine the binding constant (K_a_; K_a_ = 1/K_d_) between the Fis and i-motif DNAs using the following equation:

$$\frac{\Delta (\frac{{{\rm{I}}}_{{\rm{T}}}}{{{\rm{I}}}_{{\rm{N}}}})}{\Delta {(\frac{{{\rm{I}}}_{{\rm{T}}}}{{{\rm{I}}}_{{\rm{N}}}})}_{max}}=\frac{{{\rm{B}}}_{max}\,[{\rm{D}}{\rm{N}}{\rm{A}}]}{{{\rm{K}}}_{{\rm{d}}}+[{\rm{D}}{\rm{N}}{\rm{A}}]}$$

where B_max_ is the maximum specific binding expressed in the same units as Y-axis. K_d_ is the dissociation binding constant.

### CD spectroscopy analysis

CD spectroscopic experiments were carried out on a Jasco (J-1500) spectropolarimeter using a 1 mm path length cuvette. The CD spectrum was taken according to the protocol mentioned in Bhattacharjee *et al*.^21^. The concentration of i-motif DNA was kept constant (20 µM) while varying the concentration (20 µM, 60 µM, and 100 µM) of Fis. The composition of the buffer was 50 mM KCl, 10 mM KH_2_PO_4_, and 1 mM K_2_EDTA (pH 5.4) (25 °C).

### UV melting analysis

The thermal denaturation experiments were carried out with a model Shimadzu UV-1800 spectrophotometer equipped with a Peltier temperature controller using 1 cm path length quartz cuvette. The absorbance of 10 µM i-motif DNA at 260 nm was continuously detected from 20 °C to 90 °C at a rate of 0.5 °C/min. The buffer used here contains 50 mM KCl, 10 mM KH_2_PO_4_, and 1 mM K_2_EDTA (pH 5.4). The UV melting curves were normalised and analysed by the curve fitting using Origin Pro 8 software. The thermodynamic parameters were determined based on two-state model thermodynamics^10^.

### DNA replication

The assays were based on a previous report^10^. Briefly, fluorescence-labelled primer and template DNAs containing VEGF IM were annealed in the buffer used in the replication reaction, which consisted of 40 mM MES (pH 6.0), 8 mM MgCl_2_, 50 mMKCl, 1 μM Klenow Fragment exo- (KF exo-), 1 μM DNA, and 250 μM dNTPs with or without 100 µM Fis. After preparation of the solution, the mixtures were incubated at 37 °C. The reaction was stopped by the addition of the solution containing EDTA and formamide. Products were separated on 12% polyacrylamide gels containing 8 M urea at 70 °C for 1 h at 200 V in TBE buffer. The gel images were captured using a Fujifilm FLA-5100 fluorescent imager. The intensities of bands were analysed by NIH ImageJ software. The amounts of full-length product and stalled product at i-motifs were quantified by calculating the ratio of the intensity of each product band to the intensity of all bands. The kinetic model was applied to the two-step sequential model;

$${{\rm{P}}}_{{0}}\mathop{\to }\limits^{{{\rm{k}}}_{{\rm{s}}}}{{\rm{P}}}_{{\rm{S}}}\mathop{\to }\limits^{{{\rm{k}}}_{{\rm{f}}}}{{\rm{P}}}_{{\rm{F}}}$$

where P_0_ is the starting state of reaction, P_s_ is the state immediately after dissolving the stall by non-canonical structure is removed, P_f_ is the state after completion of replication, *k*_s_ (min^−1^) is the rate constant of dissolving the stall from reaction start by KF exo-, and *k*_f_ (min^−1^) is the rate constant of full-length product after dissolving the replication stall. Due to the relatively low stability of VEGF IM, *k*_s_ and *k*_f_ could not be separately determined. To determine the *k*_s_ value, the time course of the amount of the stalled product *A*_*s*_ was simply fitted by the exponential decay model;

$${A}_{S}={A}_{max}\{1+{e}^{(-{k}_{s}t)}\}+B$$

where *A*_max_ is the maximum amount of *A*_s_, *t* is the reaction time (min), and *B* is the background fluorescence intensity. The plot at 0.16 min was omitted for the analysis, because we could not separate the stalled product from the non-stalled product. Three independent results were analysed to evaluate rate constants by the Kaleida Graph (Synergy Software).

## Supplementary information

supplementary information.
