# Supplementary material for: Preferential targeting cancer-related i-motif DNAs by the plant flavonol fisetin for theranostics applications
Source: Sci Rep. 2020 Feb 13;10:2504. doi: 10.1038/s41598-020-59343-2 (PMC7018961; doi:10.1038/s41598-020-59343-2)
Supplement: Supplementary file 1 — supplementary information. [file 41598_2020_59343_MOESM1_ESM.pdf]

Supplementary Information

Preferential targeting cancer-related i-motif DNAs by the plant flavonol fisetin for theranostics applications

Shuntaro Takahashi[1], Snehasish Bhattacharjee[2], Saptarshi Ghosh[1], Naoki Sugimoto[1,3]* and Sudipta Bhowmik[2]*

[1] FIBER (Frontier Institute for Biomolecular Engineering Research), Konan University, 7-1-20 Minatojima-Minamimachi, Chuo-ku, Kobe 650-0047, Japan
[2] Department of Biophysics, Molecular Biology & Bioinformatics, University of Calcutta, University College of Science, 92, A.P.C. Road, Kolkata 700009, India
[3] FIRST (Graduate School of Frontiers of Innovative Research in Science and Technology), Konan University, 7-1-20 Minatojima-Minamimachi, Chuo-ku, Kobe 650-0047, Japan

* To whom correspondence should be addressed. Dr. Sudipta Bhowmik Tel: [+91 9735887221]; Email: [sbbmbg@caluniv.ac.in or su_sudipta@yahoo.co.in] Correspondence may also be addressed to Prof. Naoki Sugimoto. Tel: +81-78-303-1147; Fax: +81-78-303-1495; Email: sugimoto@konan-u.ac.jp

Figure S1:

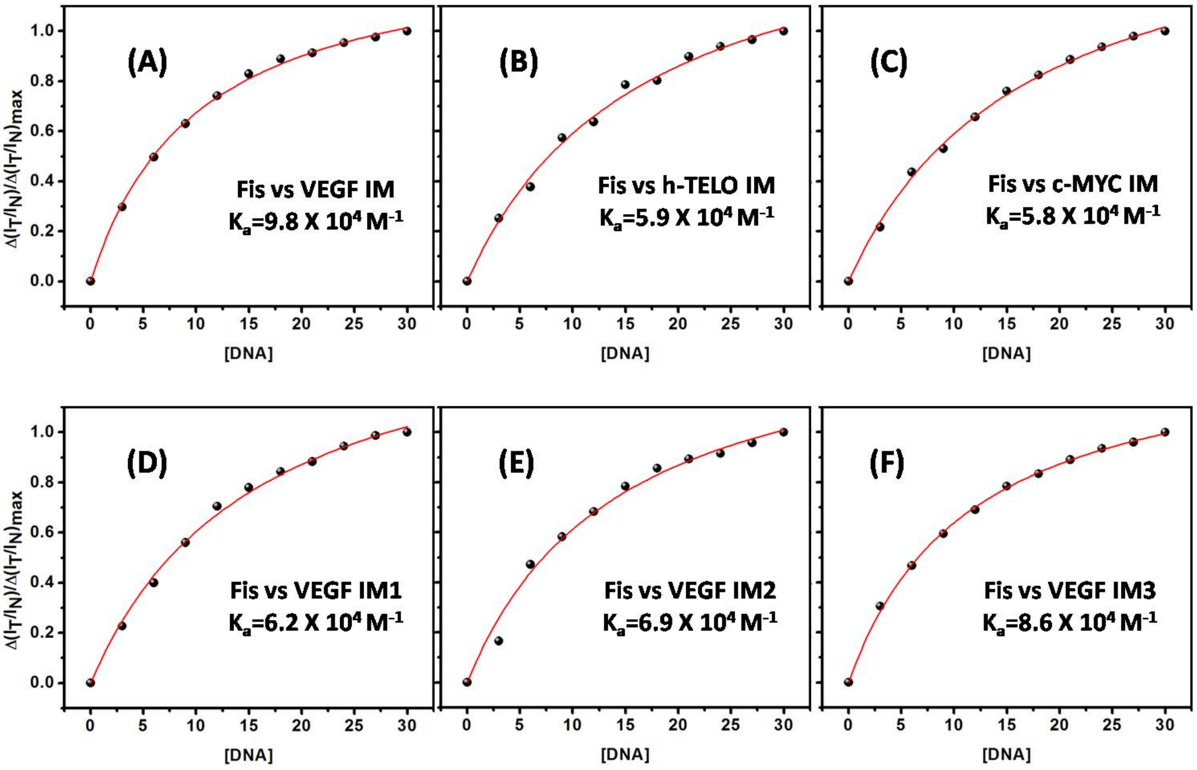

Figure S1: Binding isotherm plots to determine the affinity binding constant (K_a) from fluorescence titration experiment.

Figure S2:

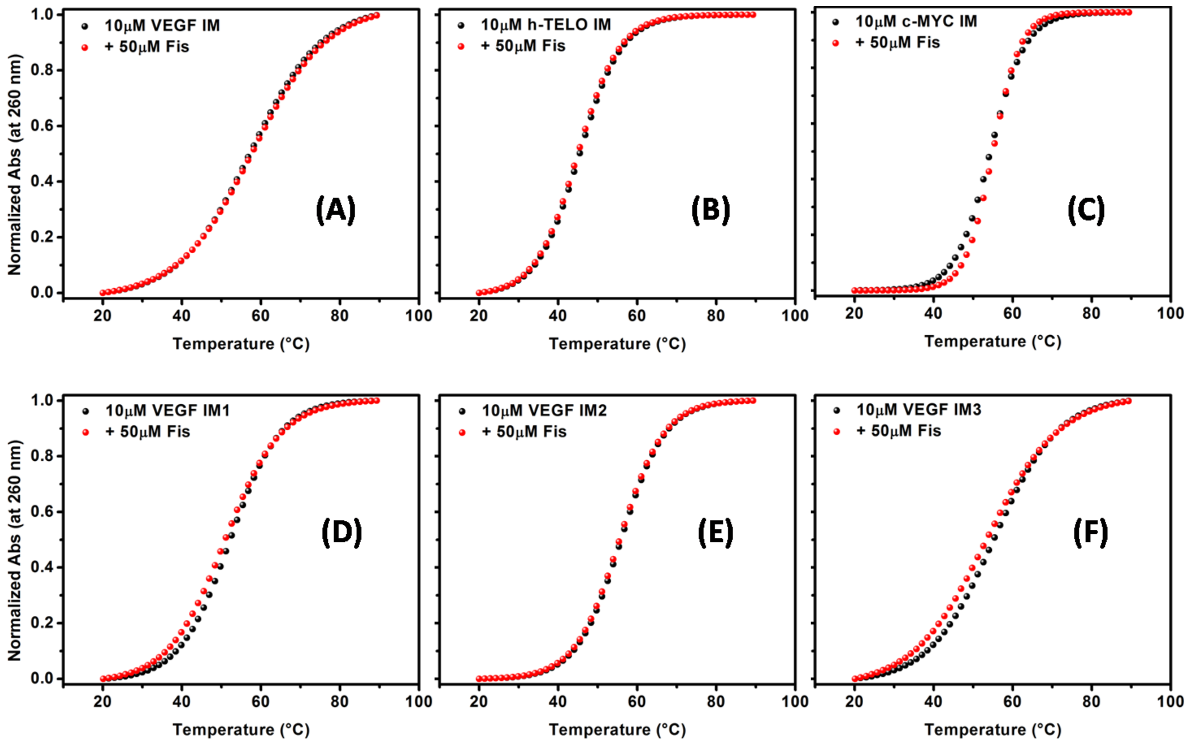

Figure S2: UV melting curves of different i-motif DNA (10 μM) structures in the absence and presence (50 μM) of Fis.

Figure S3:

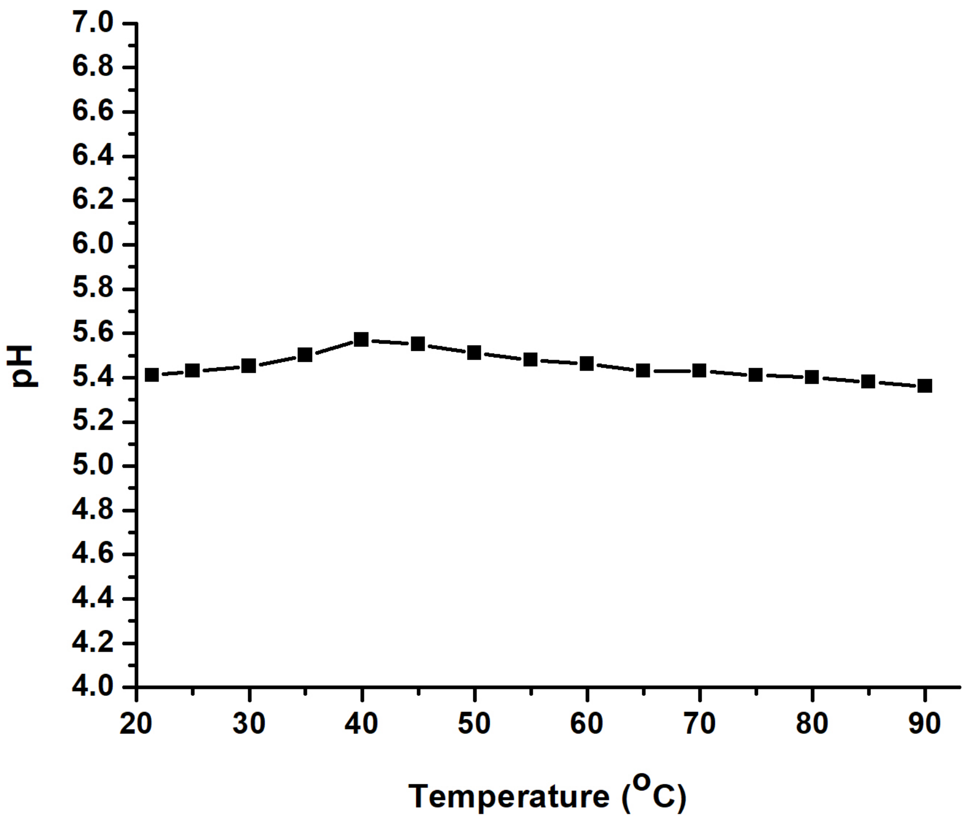

Figure S3: Monitoring the pH change of phosphate buffer (50 mM KCl, 10 mM KH_2PO_4, and 1 mM K_2EDTA; pH 5.4) over the temperature range 20 to 90°C.

Figure S4:

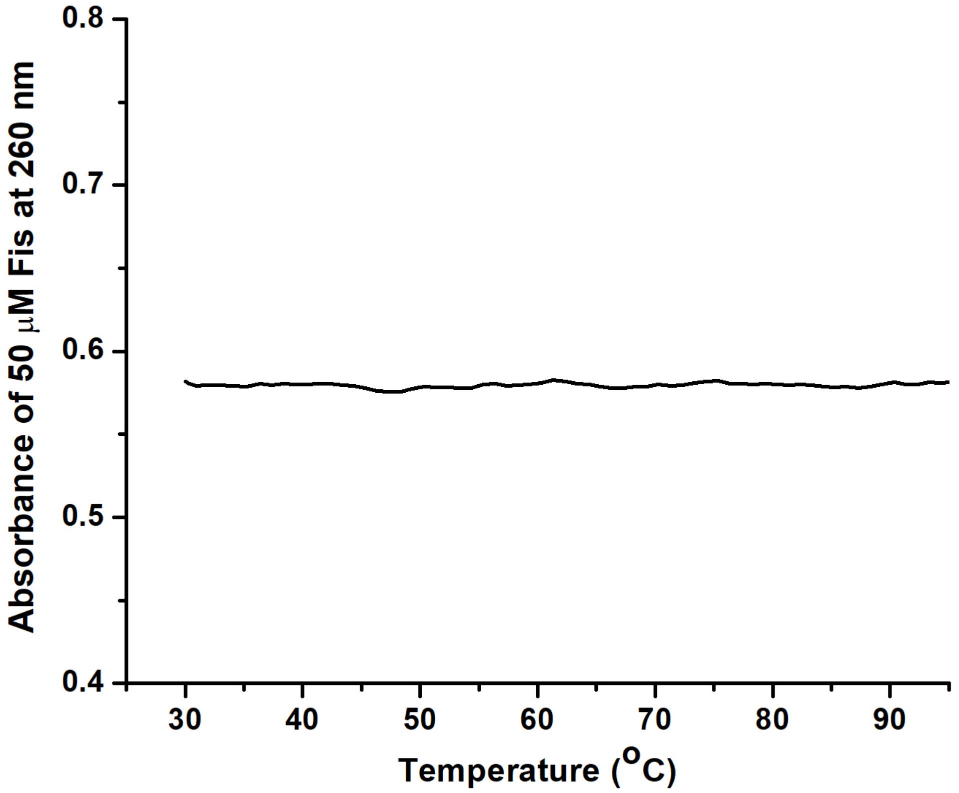

Figure S4: Monitoring the absorbance of Fis (50 μM) at 260 nm in phosphate buffer (50 mM KCl, 10 mM KH_2PO_4, and 1 mM K_2EDTA; pH 5.4) over the temperature range.

Figure S5:

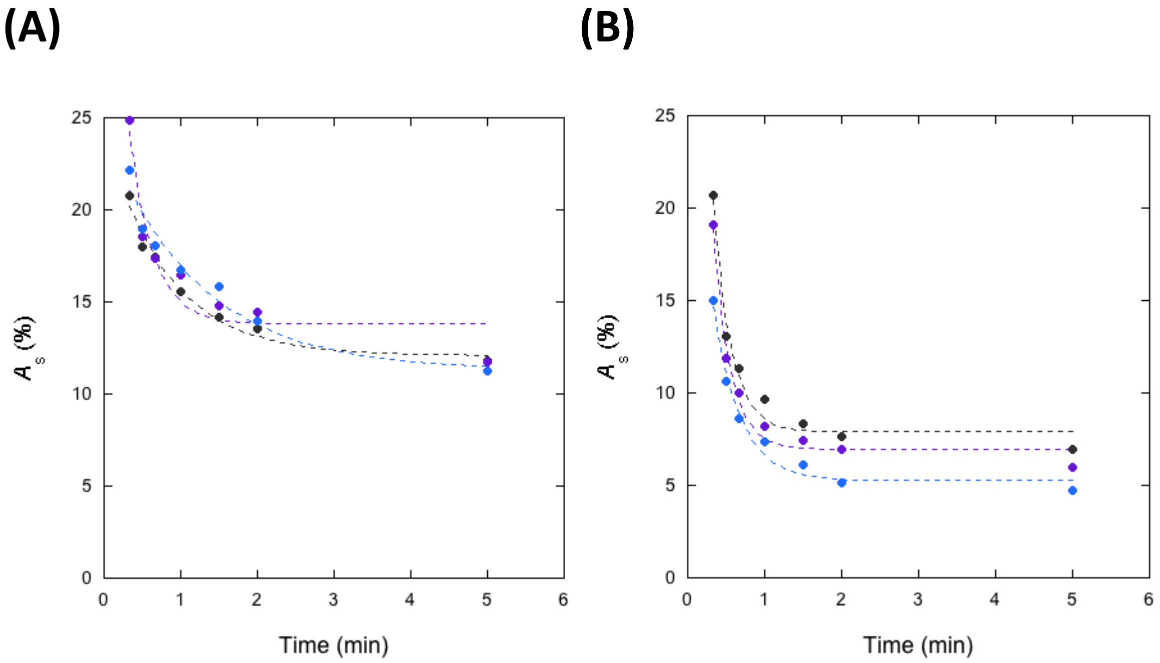

Figure S5: Analyses of resolving of stalled product in reactions of VEGF IM (A) in the absence of Fis and (B) presence of 100 µM Fis. All the plots were from three independent assays. The dotted lines indicate each fitting curve. All the replication assays were carried in 40 mM MES (pH 6.0), 50 mM KCl, 8 mM $MgCl_2$, 1 µM KF exo- , 1 µM DNAs, and 250 µM dNTPs at 37°C.

Figure S6:

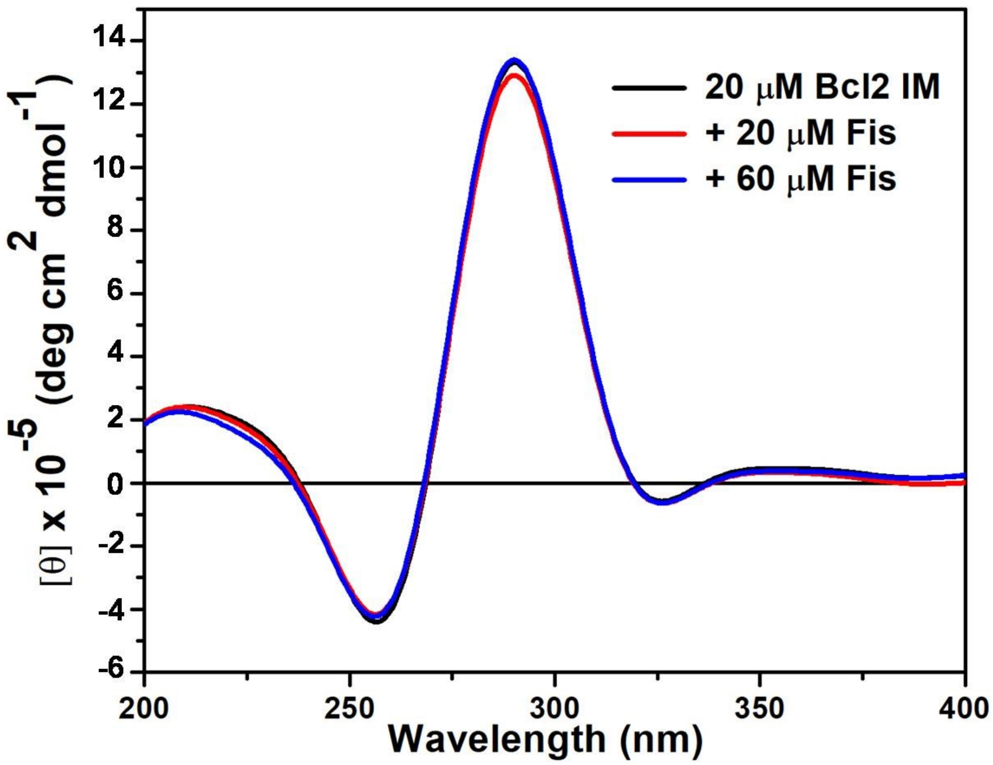

Figure S6: CD spectra of Bcl2 IM DNA in the absence (black spectrum) and presence of different concentrations of Fis. All the experiments were performed in a buffer consisting of 50 mM KCl, 10 mM KH_2PO_4, and 1 mM K_2EDTA (pH 5.4) at 25°C.
